# Selections from Foreign Journals

**Published:** 1888-10

**Authors:** 


### SELECTIONS FROM FOREIGN JOURNALS.

Two cases of Exophthalmic Goitre in Animals. By Dr. Evsaienko, of Russia, from the "*Archive of Veterinary Science*," published by the Russian Imperial State Department, St. Petersburg, June, 1888. Abstracted by Robert Formad, jr., V. M. D., Assistant Demonstrator of Normal Histology, University of Pennsylvania.

Before giving the account of these cases the translator begs leave to remark that in human medicine a large number of cases of Exophthalmic Goitre have been described in Europe as well as in this country; but the veterinary literature on this subject is very limited, in fact not even a mention of the existence of this variety of Goitre was he able to find.

In man this disease was first observed by Grave, of England, (in 1835), but has been more elaborately described by Basedow, of Germany, (in 1840), and is frequently designated under the latter name as Basedow's disease, although the name of Grave's disease has been also used. A number of other synonyms are used; the most common of these being: Stouma Exophthalmic, Cachexia Exophthalmica, Exophthalmic Goitre, Trachycardia Strumosa Exophthalmica, Morbus Basedowic, etc.

The real cause of the disease is not fully established, nor is its pathology clearly understood, and it is regarded by some as a nervous disease, by others as a dyserasic condition, still others regard it as a derangement of circulation. Some of the predisposing causes are said to be: heredity, blood diseases (chlorosis pernicious anaemia), nerve derangements, frequently being a sequella of epilepsy, chorea, etc.

Exciting causes are such as poisonous plants, fright, muscular exertion, injuries to the head and cervical region, leaching or cupping in the occipital regions of the head, etc. The climate seems to have some influence. Contrary to common Goitre, which occurs in mountainous countries, Exophthalmic Goitre is more common in the flat lands of northern Germany and Russia along the Baltic Sea-coast and is rare in France or Switzerland. Dr. Evsaienko records the following two cases: one in a mare, the other in a bitch.

First case. A small pug bitch, 7 years old, fell suddenly sick with epileptic fits, after a run on a hot day. A veterinarian was called and he found the dog in a languid, sleepy, semi-unconsious condition, without any desire to move, seeking dark places and rousing violently when touched. Temperature 38.9° C. Appetite entirely lost, but thirst excessive. The action of the heart was intensified and accompanied with increased rapidity of the pulse. No diagnosis had been made at that time. Treated with quinine, salicylate of soda, hypodermic injections of strichnia, etc., at various times during two weeks, the disease remained unchanged, but after that the fits became more violent and an eruption covered the whole body of the animal. This phenomena appearing at one time and disappearing at another, confounded the practitioner and he decided to consult with Dr. Evsaienko, who took from that time on, charge of the case. Dr. Evsaienko found the animal in a very anaemic irritable condition, motionless, without the slightest inclination to get up, and emittin; at intervals prolonged groans. The temperature registered 39.1° C. The pulse increased in frequency. The heart's action not showing any marked abnormality conducted at times a slight metallic sound, which could be heard more distinctly at the base of the heart. The thyroid gland, right side, was a little enlarged. The whole body was covered with little blotches, resembling urticaria, which were more distinct on the abdomen. They were of variable size and color, some being red, while others were pale with a blueish tint and surrounded with a red circle of hyperaemia, still others were broken in the centre and a semi-liquid material exuding, which glued together the surrounding hairs. The animal was greatly debilitated from diarrhœa that had ensued, and had total loss of appetite.

The described condition of the animal in Dr. Evsaienko's opinion resembled to a certain extent marasmus, and so the treatment was directed accordingly. In the course of a few weeks the animal improved greatly. The catarrhal condition of the intestines became controlled, the diarrhœa diminished; the rash on the skin disappeared entirely, leaving only leucodermata on the surface of the skin; the appetite improved also; the animal became livelier, and got up of his own accord and walked across the room. But this improvement did not continue for a long time and the disease returned with renewed gravity. The accelaration of the pulse and the increased action of the heart returned as before. The swelling of the thyroid gland became larger, uniform, fluctuating in character and was clearly indicative of Goitre. Both eyes were puffy, the eye-balls protruding (Exophthalmous) to its highest degree. The right eye-lid was retracted, leaving the greater part of the eye-ball uncovered; exposing the sclerotic and giving the eye an unusual lustre; on the whole resulting in a hideous, ugly appearance. The left eye-ball was still worse, and almost the whole eye-ball was protruding and uncovered by the eye-lid. The centre of the cornea of this eye was the seat of an ulceration nearly one-eighth of an inch in diameter, the conjunctiva considerably hypertrophied. The general condition of the animal became very poor; moreover, as a pernicious anaemia developed, which made the chances of recovery very doubtful. The unprotected eye-ball being constantly exposed could easily lead to inflammation of the cornea, or cause blindness from the ulceration which had affected the left cornea. Such a condition called for a prompt symptomatic treatment. Both eyes were washed with a solution of bi-chloride of mercury (1-1000) and an application of ung. potas. iodid. cum ex. belladonna to the eyelids. Three drops of eserine sulphuric (gr. 1 to oz. 1) were droped into the eyes and a compress dressing (small pad saturated with a 4 per cent. sol. salicyl. of borax) applied. It was proposed to stitch up the eye-lids together, so as to prevent injury of the cornea, but the existing ulceration of the cornea, the impossibility to keep the eye-lids in a state of quiet, led to postponement of the operation, and instead of which an injection of eserine was tried. Into the thyroid gland eight drops of tinct. iodine were injected. The animal was also given solution of potas. iodid. (gr. 11 to oz. 1) in teaspoon doses every two hours. Iodine-belladonna ointment was applied to the eye-lids and the thyroid gland. This treatment was continued for two days. But as the left eye-ball did not improve, then five drops of scrapuline sulphur. (gr. 1 to oz. 1) dropped into eye. The swelling of the eye remained unchanged. The next day it became apparent that the vision of the left eye would be lost. The pupil was not sensative to the light, the borders of the ulcerated cornea became more and more infiltrated, the clouded spot increased in size and its bottom having opaque gray color with ragged edges. The general condition of the animal improved slightly, the temperature dropped to 38.2° C. The increased heart's action and accelarated pulse became apparently quieter, and the animal eat some meat-broth after a fasting of five days. Three days later the animal was given a cold bath, after which it became so agile, as to be able to move of his own accord, although these movements were very uncertain and shaky on account of the great weakness of the animal. Injections of iodine into the thyroid gland followed by the described ointment, and application of hot poultices in the region of the throat were continued. The eyes were later washed with solution of bi-chloride of mercury (same strength as befor[illegible]), then three drops of scrapuline were put in each eye. Several days later, the indurated point in the cornea of the left eye began to clear up and to fill in with embryonal tissue. The protrusion of the eye-ball disappeared entirely. Cold baths were repeated. Likewise the eyes were washed with bi-chloride of mercury and scrapuline dropped in. There was no appreciable change in the thyroid gland. An enema was given as the animal had not evacuated his bowels for two days. The described treatment was continued, and in a month the animal made a perfect recovery. The point at which the ulceration took place, filled up entirely, leaving only a bearly appeciable cloudiness there, but enabling the animal to see with the left eye. The enlarged thyroid gland returned almost to its normal size, leaving only a thickening of the skin at that place. The abnormality of the heart's action and of the pulse disappeared entirely.

Second Case of Exophthalmic Goitre ending fatally.

A valuable bay trotting mare four years old developed sickness the day following a hard race. She lost her appetite, became very thirsty, languid and sluggish. After all the domestic (home) medicines had been tried by the trainer, a veterinarian was called in. An examination showed the presence of fever, temperature 39. 8° C., increased and deepened respiration, accelarated heart's action, accompanied with quickened pulse. The right lobe of the thyroid gland increased in size. The surrounding tissues were also swollen. Some puffiness of both eyes and conjunctiva were present. Cerebral derangement was supposed and cold water applied to the head, calomel cathartics and enema was ordered.

As time passed on and the disease, instead of responding to treatment became more aggrevated, a consultation was decided on and four veterinarians met, among whom the author was present. During two weeks standing the disease advanced considerably in gravity. Both eye balls protruded to its highest degree preventing entirely the closing of the eye. The conjunctiva was deeply congested and partly protruding. The protruding eye balls had an unusual lustre and rigid expression. The uniform enlargement of the thyroid gland was evidently due to a certain extent to the dilatation of the thyroid arteries. This was proved by the pulsation of these vessels which were distinct enough, not only to be felt but also to be seen. The contractions of the heart were increased in frequency, but did not convey any organic lesions even through the stethoscope, beyond an increased systalic sound, temperature 40. 1°C. The animal refused to take food or water. It was agreed to administer internally Potass iodide; to wash the eye with Bi-chloride of mercury (1.1000); then to apply compress dressing; to use cold water baths and inject iodine into the thyroid gland, etc. This treatment was continued for about two weeks, but the animal perished, probably from the supper-added grave attack of pernitious anæmia. Unfortunately no post mortem was made, as the practitioner in whose care the case had been was too busy at that time.

On the Action of Pilocarpin. By Army Veterinary Surgeon Maximillian in Stendal.—*Archiv fur Wissenschaftliche und Praktische Thierheilkunde. Band* 14, *Heft* 4 *to* 5.

By the use of Pilocarpin I have obtained results which, I think, have never been called to notice. I beg leave, therefore, to offer a few notes thereupon.

A large and strong horse suffered for about a year with blind staggers, which finally rendered the animal completely useless.

The death of the animal having been decided upon, I sought and obtained permission to put him under the pilocarpin treatment as an experiment.

After the first injection of 0.5 grammes in the left side of the neck, the usual symptoms appeared, though not in marked degree: the sweating especially being neither severe nor of long duration. It was therefore determined to administer a stronger dose at the end of a week. Two days after the first injection the appearances about the innoculated part were the same as immediately after the injection. A swelling which had appeared about the innoculated part at this time sunk and diffused.

After the second injection of 0.8 grammes in the other side of the neck, a swelling the size of one's fist appeared, which, as in the first case, after the second day disappeared and a repetition of the appearances. in more marked degree than after the first injection, followed, though without the sweating.

The result was very satisfactory, in fact, astonishing. The horse appears as other horses, and has become quite useful. Six weeks have passed and there has been, as yet, no recurrence of the trouble. Whether the improvement is permanent has yet to be determined. At any rate, the injection can be repeated if symptoms of the disease appear.

A. W. C.

Tuberculosis (Lymphadenoma) in the Horse. By Thomas Campbell, F. R. C. V. S., Kirkudbright. *Journal of Comparative Pathology and Therapeutics*, June, 1888.

Within the last few years I have had three cases of so-called lymph-adenoma, and as one and all of us admit the difficulty we have in diagnosing such cases, perhaps the history of one of them may be of interest to the profession. The subject was a bay Clydesdale mare.

The foreman having observed that the mare was not in the condition that might have been expected considering the quantity of good nourishing food she was eating, sent her in to my premises to be examined. I was not at home when she arrived, but one of my pupils (Mr. Bowhill), who examined her, failed to find any organic disease, and, having been told by the man who worked her that, while feeding, a great quantity of saliva came from her mouth, and that she did not eat much hay, he came to the conclusion that something was amiss with her mouth. He accordingly dressed her teeth with the rasp and ordered an astringent lotion for the mouth.

About ten days after this, Mr. Sprout, considering that the animal did not show any signs of improvement, ordered her to be sent in to me for re-examination. This was about the middle of March, 1886.

The mare looked in fairly good condition, but her coat had a dry, harsh appearance, and the old coat showed no signs of shedding. She had also a dull, listless expression, with a weak and slightly accelerated pulse. I found the brachial lymphatics very much enlarged, as also the sub-maxilliary, which showed signs of recent suppuration.

I at once came to the conclusion that the mare was suffering from some serious chronic malady, so I ordered her to be taken home and allowed to stand at perfect rest for three days, after which, I informed him I would go out to the farm and again examine her. This I did on the 16th March, my pupil (Mr. Bowhill) taking a note of the symptoms, which I have unfortunately mislaid. However, several of the important symptoms which, in addition to those already enumerated, led me to diagnose the case as one of lymph-adenoma, are still fresh in my memory. On examination per rectum, a mesenteric gland as large as a goose egg could be felt. I could not feel the spleen, but on percussing the abdominal wall I imagined I detected an abnormally dull sound, and to this I drew Mr. Bowhill's attention. In passing my hand up the inside of the thigh wall into the groin, I felt the inguinal lymphatic glands, on both sides, enormously enlarged. The skin was hard and seemed as if fixed to the ribs. The conjunctiva was yellow, although the visible mucous membranes were pale. The pulse was soft, weak, and greatly accelerated by the slightest exertion.

The appetite was fairly good, the animal eating boiled oats and bran, but not inclined to eat much hay. When not feeding she stood back in the stall stretching the halter to its full length. The bowels were very irregular, at one time constipated, the next time the reverse. She walked well on level ground, but when made to walk down hill she showed signs of distress, would breathe rapidly, and cough; the cough evidently being of a painful character.

I gave my client an unfavorable prognosis, but, at the same time, I said that if he had no objection to the expense, I would try a course of medicine. To this he replied that he would leave the case entirely in my hands, so I resolved to give a course of iodide of potassium, followed up with powders of quinine, iron and nux vomica, which she took in her food without any difficulty. No good results being observed to follow this treatment, I ultimately stopped it and turned her out to good pasture to "take her chance."

About a fortnight or three weeks after this I had a visit from my friend,

Mr. Banham, of Cambridge, and as I thought the case would interest him, I drove him out to see it. He did not disagree with me as to the nature of the malady, but remarked that it was difficult to diagnose such a case in one visit. I had examined the blood previously, and, at his suggestion, I again examined it but failed on both occasions to detect the slightest difference between the blood of this subject and normal blood. Two days after this the mare was found dead in the field, and I was made acquainted with this soon afterwards. I lost no time in making a post-mortem examination, and from the lesions present, I feel surprised that the animal had existed for such a long time. All the important organs were affected, some of the mesenteric glands being nearly a pound in weight. The spleen was an enormous size, and very little of the normal structure remained, its place being occupied by a multitude of tumors, varying in size from that of a hazel nut to that of a cocoanut. Numerous small tumors were studded throughout the structure of the liver, the lungs being similarly affected.

The muscular tissue had a pale appearance, and the sub-maxillary glands were undergoing degeneration.

Thinking that the case might be of some pathological importance, I sent the numerous specimens to the Dick Veterinary College, Edinburgh, and as the readers of the *Journal of Comparative Pathology* are aware, Professor McFadyean found on microscopic examination of the new growths that they were of a tubercular nature, and contained the specific bacilli in enormous number. After reading the article written by Professor M'Fadyean, which appeared in last issue of the JOURNAL, I consider myself amply repaid for all the interest and trouble I took in the case.

A. W. C.

TUBERCULOSIS IN THE HORSE. By John M'Fadyean, M. B., B. Sc. *Journal of Comparative Pathology and Therapeutics*, March 1888.

The occurrence of true tubercular disease in the equine species has not, so far as I am aware, been described by British veterinary writers. Indeed, it is a pretty general opinion among the members of the profession in this country that the horse has a very decided, if not an absolute immunity from tuberculosis. The late Professor Robertson, in his work on Equine Medicine says: "The nearest approach to true miliary tubercle in the organ of the horse is probably the diffuse granulation masses found in the lungs and other parts of the pulmonary tissues in glanders,"

Nevertheless, it is certain that horses occasionally do contract a true tuberculosis. Prior to the year 1882, any such statement as that might have been contested, for the definition of the term tubercle rested chiefly upon anatomical characters; and different authorities were by no means agreed as to what morbid growths and products were to be classed as tuberculous, and what others were to be excluded from the list. In the year mentioned, however, Koch, by his discovery of the specific bacillus, gave to the application of the term *tuberculosis* an absolute precision. By that word we must now understand a morbid process caused by a specific micro-parasite —the *bacillus tuberculosis*.

It is true that the histological characters of the tubercles and other types of new tissue formed under the influence of the parasite, are so definite, that with a little experience one can, in most cases, be almost certain whether any new growth under examination is or is not tubercular, using the word in its restricted sense.

Nevertheless, the final and absolute proof in every case must rest upon our ability to demonstrate in the diseased parts, the presence of the specific bacilli.

In the month of February, 1886, there came into my hands portions of the spleen and lungs of a mare that died in the practice of Mr. Joseph Donald, F. R. C. V. S., Wigton, Cumberland.

Before proceeding to describe the naked eye and microscopic character of the lesions present in these organs, I may briefly detail the clinical history of the case, which Mr. Donald has been good enough to communicate to me from a record made at the time when the mare was under his care.

As early as the winter 1884–85, it had been observed that this mare had become unthrifty, fed badly, and got into low condition. At the time, however, this was attributed by the owner to overwork. When the farm work was finished, early in the summer of 1885, the mare was turned out to grass, and subsequently she appeared to thrive very well up till the end of the year. At this time the mare again commenced to fall off in condition and Mr. Donald was called in to see her in January, 1886. The chief symptoms noted were loss of appetite, a dull appearance, great thirst and profuse urination. Mr. Donald, considering that he had to deal with a case of *diabetes insipidus* prescribed iodine combined with tonics. Under this treatment the diabetic symptoms, to a great extent, disappeared, and the appetite improved. This improvement, however, was of short duration; the appetite again became bad, the general appearance became worse, and emaciation, with loss of strength, rapidly set in, Now, also, for the first time, the mare gave evidence of lung affection, and Mr. Donald gave an unfavorable prognosis. The mare died a few days later. The first lung symptoms were detected on the 4th February, six days prior to death. The highest temperature recorded during the course of the illness was 103° F.

The organs sent to the Veterinary College by Mr. Donald presented the following characteristics. The spleen contained several tumors, the largest being about the size of a turkey's egg. These tumors were greyish white in color, and the larger masses were softened and caseous centrally and partially calcified.

One or two smaller growths, apparantly greatly enlarged lymphatic glands, were present in the neighborhood of the bilus. The pieces of lung presented a very unusual appearance, the pulmonary tissue being densely and uniformly infiltrated with a new growth of a minute milliary character. The density of the lung tissue was much increased ; it cut more firmly than normal, but consolidation did not appear to be complete in any part.

The microscopic examination of this lung, after it had been hardened, revealed what may be designated as a typical tubercular formation.

The new growth was for the most part small celled, but numerous giant cells were present in the tubercles. Some of the larger foci were becoming caseous towards the center. The spleenic growths were recent, presented a somewhat similar structure, but the process there was evidently of a more chronic character, as evidenced by the more extensive caseation and commencing calcification. I was now in little doubt that I had to deal with a case of tuberculosis. I therefore proceeded to stain sections in order to discover the tubercle bacilli. On this head, suffice it to say that by the appropriate methods I was able to demonstrate these organisms in enormous numbers. Some of the giant cells contained the bacilli so numerously that in decolorized sections they stood out distinctly under a low power of the microscope as deeply stained bodies. A higher magnification showed that this color was due to stained bacilli in their interior.

A short time after the receipt of Mr. Donald's specimens, there were sent to the Veterinary College, the spleen and part of the lungs of a horse that died in the practice of Mr. Campbell, F. R. C. V. S., Kircudbright.

Here, again, the spleen contained numerous tumors, which differed from present, in the previous case, in their greater size—the longest being as big as an infant's head, and in being more intensively calcified towards the center. Large thrombi were present in the spleenic veins. The lung in this case presented a lesion identical with that presented in Mr. Donald's specimen.

The microscopic examination of the new growths in this case revealed a condition identical with that already described, viz: a characteristic tubercular structure with great numbers of the specific bacilli.

Although these are, we believe, the first cases of equine tuberculosis recorded in this country, it is possible that the disease is not so rare as might be supposed. The occurrence of tuberculosis in the horse is well known to continental veterinary surgeons, and Koch, in his classical essay on the etiology of tuberculosis, states that he had the opportunity to investigate four cases. It appears to me not improbable that cases of tubercle in the horse may be overlooked, the spleenic tumors being set down as instances of so-called lymph-adenoma. This would be particularly liable to occur where the disease is confined to the abdominal viscera.

In the majority of cases occurring in the horse, the infection appears to reach the system from the intestine, at least, it manifests itself in the first instance in connection with the spleen or abdominal lymphatic glands.

Here the disease may run a chronic course, but eventually the tubercular virus is apt to burst into a vein, and milliary tuberculosis of the lung then rapidly ensues. It is important to remember that in order to distinguish between true tuberculosis growths in the spleen and lymph-adenoid, or other non-tubercular tumors, microscopic characters are not alone to be relied upon.

